# Cardiac surgery in 260 octogenarians: a case series

**DOI:** 10.1186/1471-2253-15-15

**Published:** 2015-01-26

**Authors:** Anna Mara Scandroglio, Gabriele Finco, Marina Pieri, Roberto Ascari, Maria Grazia Calabrò, Daiana Taddeo, Francesca Isella, Annalisa Franco, Mario Musu, Giovanni Landoni, Ottavio Alfieri, Alberto Zangrillo

**Affiliations:** Department of Anesthesia and Intensive Care, IRCCS San Raffaele Scientific Institute, Via Olgettina 60, 20132 Milan, Italy; Department of Medical Sciences “M. Aresu”, Cagliari University, Cagliari, Italy; Department of Cardiac Surgery, IRCCS San Raffaele Scientific Institute, Milan, Italy; Vita-Salute San Raffaele University, Milan, Italy

**Keywords:** Octogenarian, Cardiac surgery, Mortality, Complications, Anesthesia, Intensive care

## Abstract

**Background:**

The elderly undergo cardiac surgery more and more frequently, often present multiple comorbidities, assume chronic therapies, and present a unique physiology. Aim of our study was to analyze the experience of a referral cardiac surgery center with all types of cardiac surgery interventions performed in patients ≥80 years old over a six years’ period.

**Methods:**

A retrospective observational study performed in a university hospital. 260 patients were included in the study (3.5% of the patients undergoing cardiac surgery in the study period).

**Results:**

Mean age was 82 ± 1.8 years. Eighty-five percent of patients underwent elective surgery, 15% unplanned surgery and 4.2% redo surgery. Intervention for aortic valve pathology and coronary artery bypass grafting were performed in 51% and 46% of the patients, respectively. Interventions involving the mitral valve were the 26% of the total, those on the tricuspid valve were 13% and those on the ascending aortic arch the 9.6%. Postoperative low output syndrome was identified in 44 patients (17%). Mortality was 3.9% and most of the patients (91%) were discharged from hospital in good clinical conditions. Hospital mortality was lower in planned vs unplanned surgery: 3.8% vs 14% respectively. Chronic obstructive pulmonary disease (OR 9.106, CI 2.275 – 36.450) was the unique independent predictor of mortality.

**Conclusions:**

Clinicians should be aware that cardiac surgery can be safely performed at all ages, that risk stratification is mandatory and that hemodynamic treatment to avoid complications is expected.

**Electronic supplementary material:**

The online version of this article (doi:10.1186/1471-2253-15-15) contains supplementary material, which is available to authorized users.

## Background

The elderly (i.e. patients aged 80 years or more) represents an increasing percentage of the patients candidate to undergo major surgery. These patients are different from other age groups, as they often present multiple comorbidities, assume chronic therapies, and present a unique physiology in terms of respiratory, cardiovascular, and metabolic systems [1, 2].

Cardiac surgery is increasingly performed in old patients too, with many interventions being performed in emergent or urgent conditions, and not only as planned surgery [3]. The impact of surgery altogether (extent of surgery, use of cardiopulmonary bypass (CPB), prolonged general anesthesia, hemodynamic instability) and the admission to the intensive care unit (ICU) following surgery may have a detrimental effect on the outcome of old people as compared to younger patients. Despite these strong important differences, the most appropriate clinical management for this subset of patients has never been determined, being left mainly to physicians’ clinical decision.

Furthermore, these patients utilize an increasing portion of ICU capacities, provoking economic and ethical concerns: is this a reasonable burden in terms of patients’ outcome and quality of life? [4].

Mortality rate in cardiac surgery performed in octogenarians was reported to be very high few decades ago, approaching the 24% in 1988 [3], and 15.7% in 1991 [5], but recent medical and technologic innovations may have reduced the mortality risk. Octogenarians surely represents a sicker population at increased risk, but nevertheless many of them undergo cardiac surgery safely.

The aim of our study was to analyze the experience of a national referral cardiac surgery center with all types of cardiac surgery interventions performed in old patients (i.e. ≥80 years old), in recent years and to evaluate outcomes.

## Methods

The study was conceived in accordance with the Declaration of Helsinki and its amendments. With approval of local ethical committee (“OSR Ethical Committee”) and patients’ written consent we collected data from all patients aged 80 years old or more who underwent cardiac surgery at San Raffaele Scientific Institute over a six years’ period. No specific written consent was obtained for this retrospective observational study since all patients’ data were anonymized and de-identified prior to analysis.

Patients were admitted to the cardiac surgery ward before the operation (routine preoperative evaluation is reported in Additional file 1: Table S5), underwent cardiac surgery under general anesthesia and were transferred to the ICU after surgery. All patients received standard premedication (morphine 0.1 mg/kg s.c. and scopolamine 0.25 mg i.m. one hour before surgery) and general anesthesia (induction with an intravenous bolus of propofol, fentanyl and muscle relaxant, maintenance with fentanyl, muscle relaxants and with halogenates and/or propofol). All patients received an intraoperative infusion of tranexamic acid: 1 g in 20 minutes followed by a 400 mg/h infusion. Moderate hypothermia (32-34°C) was maintained during CPB and myocardial perfusion during aortic cross clamping was obtained by antegrade and/or retrograde cold cardioplegia. Activated clotting time (ACT) was maintained greater than 480 seconds for CPB, heparin (starting dose = 3 mg/kg) was reversed with protamine in a 1 to 1 ratio. The mean arterial target pression was 60 mmHg during CPB and at least 65 mm Hg after CPB.

After surgery, patients were transferred to the ICU under sedation with propofol. Weaning from the ventilator was started as soon as hemodynamic stability was confirmed with no major bleeding, normothermia, and an adequate level of consciousness and pain control were achieved. Standard intravenous therapy included intravenous antibiotic prophylaxis with cephazolin for 72 hours, hydration, antiacids and diuretics as well as inotropes and devices as the hemodynamic conditions required.

Transfer out of the ICU was considered in the presence of peripheral oxygen hemoglobin saturation (SpO2) ≥94% at an inspired fraction of oxygen (FiO2) ≤0.5 by facemask, adequate cardiac stability with no major arrhythmias, chest tube drainage <50 ml/h, urine output >0.5 ml/kg/h, no intravenous inotropic or vasopressor therapy in excess of dopamine 5 μg/kg/min, and no seizure activity.

Criteria for hospital discharge to undergo rehabilitation program included hemodynamic and cardiac rhythm stability, the absence of incision site’s infection, an afebrile condition, normal bowel movement, independent ambulation and feeding.

Demographics and clinical characteristics were collected electronically together with preoperative, intraoperative, and postoperative data.

Myocardial infarction was defined as suggested by the Consensus Conference for the Universal Definition of Myocardial Infarction [6]. Arterial hypotension (systolic blood pressure <90 mmHg) with signs of end-organ hypoperfusion (i.e. decreased urine output, lactic acidosis) despite adequate fluid replacement, with impairment of cardiac function and normal or high peripheral vascular resistance, was defined as postoperative low output syndrome. Complete hemodynamic monitoring was guaranteed for each patients on the basis of clinical needs. Cardiogenic shock was defined according to the IABP Shock Trial [7]. Low cardiac output syndrome (LCOS) was defined as a decrease in cardiac output due to transient myocardial dysfunction and included cardiogenic shock as per the IABP shock trial definition [7].

Acute kidney injury (AKI) was defined as a 50% or 100% increase in serum creatinine value [8]. Creatinine clearance was calculated with the Cockroft-Gault equation [9] and the ACEF score was also calculated [10]. We defined as “redo” a patient who had already undergone sternotomy for cardiac surgery once in the past. “Postoperative atrial fibrillation” was defined as abrupt de novo onset of atrial fibrillation requiring either pharmacological or electrical cardioversion after cardiac surgery and during the ICU stay in a patient with no preexisting history of atrial fibrillation. Type I neurologic damage was defined as: death due to stroke or hypoxic encephalopathy, new nonfatal stroke, or transient ischemic attack (TIA), or stupor or coma at the time of discharge. Type II neurologic damage was defined as: a new deterioration in intellectual function, confusion, agitation, disorientation, memory deficit, or a nonmetabolic seizure without evidence of focal injury.

Data were stored electronically and analyzed by use of SAS (release 9.2 by SAS Institute Inc. Cary, NC, USA). Categorical variables are reported as numbers (percent), whereas continuous variables are expressed as mean ± standard deviation or as median (interquartile range) according to the Kolmogorov-Smirnov test. Fisher test was used to calculate p-values between 2 groups for categorical variables. Multiple Logistic regression was used to identify independent predictors of mortality. A stepwise selection method was used.

## Results

During the study period, 260 (3.5%) out of 7,357 patients scheduled to undergo cardiac surgery were aged 80 or more and were included in the study. Baseline characteristics of the study population are shown in Table 1.

**Table 1:** **Baseline characteristics**

Variable	Value
Gender (male), n	130 (50%)
Age, y	82 ± 1.8
Height, cm	166 ± 8.5
Weight, kg	68 ± 12.1
BMI	24.8 ± 3.6
Comorbidity	
> COPD, n	58 (22%)
> Preoperative EF,%	53.5 ± 10.8
> Preoperative EF ≤40%, n	41 (17%)
> Preoperative EF ≤30%, n	14 (5.9%)
> Peripheral vasculopathy, n	86 (33%)
> Hypertension, n	146 (56%)
> Type II Diabetes, n	32 (12%)
> Carotid stenosis, n	43 (17%)
> Chronic pulmonary disease, n	39 (15%)
> Angina, n	95 (15%)
> Previous AMI, n	38 (15%)
> Previous TIA or stroke, n	115 (44%)
> Previous vascular surgery, n	16 (6.15%)
> Euroscore standard	8.3 ± 2.3
> Endocarditis	5 (1.9%)
> Chronic renal failure, n	32 (12%)
> Dialysis, n	0 (0%)
Charlson Comorbidity Index	6 ± 1.3
NYHA class	
> I, n	15 (8.4%)
> II, n	82 (46%)
> III, n	74 (42%)
> IV, n	7 (4%)
Timing of surgery	
> Emergency, n	5 (2%)
> Urgency, n	33 (13%)
> Planned, n	222 (85%)
Redo, n	11 (4.2%)
Preoperative IABP, n	5 (1.9%)
Chronic Therapy	
> Antiplatelets, n	97 (37%)
> Diuretics, n	141 (54%)
> Betablockers, n	95 (37%)
> Antibiotics, n	5 (1.9%)
> Ca-channels antagonists, n	55 (21%)
> Nitrates, n	66 (25%)
> ACE- inhibitors, n	151 (58%)
> Oral anticoagulants, n	111 (14%)
> Heparin, n	35 (13%)
Bilirubine, mg/dl	0.8 ± 0.4
Creatinine, mg/dl	1.06 ± 0.4

Data are expressed as mean ± standard deviation or number (percentage).

BMI – body mass index; COPD – chronic obstructive pulmonary disease; EF – ejection fraction; AMI – acute myocardial infarction; TIA – transient ischemic attack; NYHA – New York Heart Association; CPB – cardiopulmonary bypass; IABP – intraaortic balloon pump.

Mean age was 82 ± 1.8 years, and 50% of patients were male. Forty-six percent of patients were in the functional NYHA class II, 42% were in class III, 8.4% in class I, and 4% in class IV. Preoperative ejection fraction (EF) was 53.5 ± 10.8%; 17% of patients presented an EF < 40% and 5.9% an EF < 30%, respectively. ACEF score was 1.7 ± 0.5. Despite nearly normal serum creatinine values (1.06 ± 0.4 mg/dl), creatinine clearance was impaired (50 ± 16 ml/h).

Eighty-five percent of patients underwent elective surgery, with 15% received urgent or emergent surgery (i.e. unplanned surgery); 1.9% of patients had preoperative mechanical circulatory support with intraaortic balloon pump counterpulsation. Redo surgery was performed in 4.2% of patients.

Data concerning surgical intervention performed are shown in Table 2. Intervention for aortic valve pathology and coronary artery bypass grafting (CABG) were the most frequent operations, being performed in 51% and 46% of the patients, respectively. Twenty-five patients underwent urgent or emergent CABG surgery (21%). Interventions involving the mitral valve were 26% of the total, those on the tricuspid valve were 13%, and those on the ascending aortic arch the 9.6%. Intraoperative and postoperative data are presented in Table 3.

**Table 2:** **Surgical interventions**

Variable	Value
CABG, n of patients	119 (46%)
> Isolated CABG, n of patients	63 (24%)
> Off pump CABG surgery, n	24 (9.2%)
Mitral valve surgery, n	67 (26%)
> Isolated mitral valve surgery, n	19 (7.3%)
> Mitral valve replacement, n	29 (11%)
> Mitral valve repair, n	38 (15%)
Aortic valve surgery, n	132 (51%)
> Isolated aortic valve surgery, n	71 (27%)
> Aortic valve replacement, n	131 (50%)
> Aortic valve repair, n	1 (0.38%)
Tricuspid valve surgery, n	34 (13%)
> Isolated tricuspid valve surgery, n	1 (0.38%)
> Tricuspid valve replacement, n	1 (0.38%)
> Tricuspid valve repair, n	33 (13%)
Ascending aorta surgery, n	25 (9.6%)
> Isolated ascending aorta surgery, n	2 (0.77%)

Data are expressed as number (percentage).

CABG – coronary artery bypass graft.

**Table 3:** **Intraoperative and postoperative data**

Variable	Frequency
CPB, n	236 (91%)
Duration of aortic clamping, min	61 ± 22
Duration of CPB, min	80 ± 26
Bleeding in the first 12 postoperative hours, ml	240 (170–350)
Total postoperative bleeding, ml	360 (220–530)
Creatinine peak, mg/dl	1.2 ± 0.6
Serum creatinine increase ≥50%, n	15 (5.8%)
Serum creatinine increase ≥100%, n	6 (2.3%)
Renal replacement therapy, n	8 (3.1%)
Patients receiving hemoderivates, n	110 (42%)
Red Blood Cell transfusions, n of units per patient	1.3 ± 2.34
Fresh frozen plasma transfusions, n of units per patient	0.4 ± 1.34
Platelets transfusions, n of units per patient	0.3 ± 1.58
Neurologic damage type 1, n	2 (0.77%)
Neurologic damage type 2, n	7 (2.7%)
Postoperative AMI, n	4 (1.5%)
Troponine peak, ng/ml	7.3 (4.2 - 13)
Atrial fibrillation, n	70 (27%)
Duration of mechanical ventilation, hours	15 (12–20)
Severe pulmonary dysfunction, n	5 (1.9%)
Mild pulmonary dysfunction, n	16 (6.2%)
Tracheostomy, n	7 (2.7%)
Need for re-intubation, n	6 (2.3%)
Low cardiac output syndrome, n	44 (17%)
Cardiogenic shock, n	9 (3.5%)
Inotropes for more than 48 hours, n	36 (14%)
Sepsis, n	4 (1.5%)
Mediastinitis, n	0 (0%)

Data are expressed as median (interquartile), mean ± standard deviation or number (percentage).

CPB – cardiopulmonary bypass; IABP – intraaortic balloon pump; AMI – acute myocardial infarction; ICU – intensive care unit.

Ninety-one percent of the interventions were performed under CPB and cardioplegic circulatory arrest. Mean duration of aortic clamping was 61 ± 22 minutes, and duration of cardiopulmonary bypass was 80 ± 26 minutes. A relevant portion of patients (42%) required inotropic support for CPB weaning.

Hospital mortality was 3.9%, compared to the 2.2% mortality rate observed in the 7,357 patients of all ages undergone cardiac surgery at the same institution in the same study period. Overall, mortality was lower in planned surgery compared to unplanned surgery: 2.7% vs 11% respectively.

Duration of ICU stay and hospital stay were 2 (1–4) days and 6 (5–9) days (Table 4). Postoperative bleeding from chest tubes was 240 (170–350) ml in the first 12 postoperative hours. Forty-two percent of patients received transfusions during the perioperative period.

**Table 4:** **Outcomes**

Variable	Frequency/Mean	95% CI
Hospital mortality, n	10 (3.9%)	2.1% - 6.9%
ICU stay, days	4 ± 8	3.07 - 5.2
Hospital stay, days	10 ± 11.2	8.36 - 11.11
**Clinical condition at hospital discharge**		
> Dead, n	10 (3.9%)	2.1% - 6.9%
> Extremely poor, n	2 (0.77%)	0.21% - 2.95%
> Poor, n	11 (4.2%)	2.4% - 7.4%
> Good, n	237 (91%)	87% - 94%

Data are expressed as mean ± standard deviation or number (percentage).

ICU – Intensive Care Unit.

Postoperative low output syndrome was identified in 44 patients (17%), cardiogenic shock in 9 (3.5%) and 36 patients (14%) had treatment with inotropes lasting more than 48 hours.

Four (1.5%) patients had acute myocardial infarction in the ICU, with median peak troponin value being 7.3 (4.2 - 13) ng/ml. Twenty-seven percent of patients had at least one episode of atrial fibrillation requiring pharmacological or electrical cardioversion.

Duration of mechanical ventilation was 15 (12–20) hours. Six patients (2.3%) required re-intubation due to respiratory failure after weaning from mechanical ventilation. Seven patients (2.7%) had prolonged mechanical ventilation and received tracheostomy. Acute renal failure (Table 3) occurred in 11.2% of patients, and 3.1% required renal replacement therapy. Type 2 neurologic damage was more frequent than type 1 damage: 2.7 vs 0.77% respectively. The great majority of patients (91%) was discharged from hospital in good clinical conditions.

Patients were further compared according to the gender (i.e. male versus female). Although many differences exist between genders in baseline and perioperative data, notably, no difference was found in the outcomes. (see Additional file 1 for more details).

Only one preoperative independent predictor of mortality was identified in the overall study population: preoperative chronic obstructive pulmonary disease (COPD) (OR 9.106, with 95% confidence limits 2.275 – 36.450).

## Discussion

This is one of the largest experience reported in literature by a single center on cardiac surgery performed in octogenarians, which represent the fastest growing patients’ group candidate to surgery. Typical pathophysiological changes are present in this population. Nonetheless, since individuals vary greatly in the rate at which their organs decline physiological more than chronological age should be considered in the evaluations of the patients candidate to undergo cardiac surgery [1]. Impairment of organ function manifests at different levels. Cardiovascular system is strongly affected: the heart, differently from other organs, does not atrophy with aging, but may become thicker and larger. The prevalence of diastolic dysfunction is very high, and cardiac index may be reduced [2]. Our data indeed show a preoperative ejection fraction ≤ 40% in 17% of patients and a preoperative ejection fraction ≤ 30% in 5.9% of cases, but the presence of diastolic dysfunction might be even higher. Furthermore, a deterioration in kidney function can also be observed and estimated with calculation of glomerular filtration rate, even if little changes in creatinine plasmatic value often occur [3]. In the present study, despite nearly normal serum creatinine values (1.06 ± 0.4 mg/dl), the calculation of creatinine clearance (50 ± 16 ml/h) disclosed the presence of kidney function impairment. Respiratory, hepatic, metabolic systems show a decline too, and neurologic impairment is also commonly observed. For these reasons only a minority of elderly symptomatic patients are referred for surgical treatment because the operative risk is considered too high. Not surprisingly, this fragile population of patients are at higher risk when operated, but although the mortality rate is higher that 1-3% usually observed in elective cardiac surgery in the overall population, we observed an acceptable 3.9% rate in this specific subset of high risk patients. Forty-two percent of patients received transfusions during the perioperative period confirming that advanced age still represents a high risk category for transfusion, according to the Update to the Society of Thoracic Surgeons and the Society of Cardiovascular Anesthesiologist Blood Conservation Clinical Practice Guidelines [11].

The long duration of mechanical ventilation that we recorded (i.e. 15 (12–20) hours) has multifactorial explanations including the long half-life of fentanyl, pre-existing pulmonary comorbidities, and perioperative factors and complications such as hemodynamic instability, sepsis, and pneumonia. Notably, preoperative COPD was the only preoperative independent mortality predictor in this population and it had an high prevalence in this elderly population.

Recently some studies addressed the issue of performing cardiac surgery in the old patients: the data published by Bridgest [12] on coronary surgery and by Biancari [13] on mitral valve surgery show a trend towards a reduction in mortality far from that of the nineties. In that period 80 year old patients undergoing heart surgery were reported to have a 24% mortality rate in the far 1988 [4] and 15.7% by Freeman and colleagues in 1991 [3].

In the last 2 decades, extraordinary technologic developments and evolution of knowledge has led to a reduction in mortality. Such developments include improvements in surgical technique, CPB technology and circuits, the management of cardioplegic circulation, transfusions policy, hemodynamic and coagulation monitoring, post-cardiac surgery care (including mechanical circulatory support and fluid therapy). Clinicians share the common idea that who is nowadays candidate to CABG surgery is older and sicker than in the past (more comorbidities, advanced multidistrectual vasculopathy), but a decreasing mortality rate has been reported in literature for coronary artery bypass surgery [5, 14]. This is a very important topic as coronary artery bypass surgery is among the most frequent interventions performed in our population of octogenarians, accounting for 46% of the total amount. Bridgest et al. published a very interesting study on a very large number of patients [12]. They reported data from The Society of Thoracic Surgeons National Database with 59,576 patients aged 80 who underwent cardiac surgery from 1997 to 2000. In the CABG group of those aged between 80 and 89 the mortality was 7.1%. Notably, mortality rate in CABG surgery was 3.4% in our study population, which is lower than mortality of mitral valve and aortic valve surgery in the same population (6% and 3.8%, respectively).

In their conclusion, Bridgest et al. [12] stressed the concept that, thank to a careful selection, the old patients candidate to CABG have a lower risk, similar to that of younger patients.

We strongly agree that such an approach or patients’ risk stratification may help to identify those patients with good preoperative status for whom age alone should not be considered a contraindication to surgery.

There are two main open issues regarding CABG patients: one is the issue of the most critical old CABG-patients who reach the operating theater in unstable hemodynamic condition requiring mechanical circulatory support (5 patients in our case series had preoperative IABP, of whom 3 underwent CABG), and the other one is off-pump versus on-pump CABG surgery. Theoretically off-pump surgery may present some advantages compared to on-pump cardiac surgery, as systemic inflammation, myocardial injury, and cerebral injury may be strongly reduced without CPB [15, 16] and such benefits may be relevant in patients with precarious homeostasis as the elderly. Minimally invasive surgery for a single vessel bypass might reduce even more postoperative complications. Besides, off-pump surgery was shown to be associated with less postoperative neurologic complications compared to CPB surgery [17, 18]. Mortality in aortic valve surgery is 3.8%, which seems low considering the characteristics of these patients who frequently manifest low output postoperative syndrome because of the anatomic changes of the heart secondary to the pathology, not always easy to be managed. This case series doesn’t take in account the percutaneous procedure of aortic valve replacement (TAVI). In our center this program of TAVI was still at the beginning in the period of the present data collection and reserved to the more complex patients unsuitable for surgery (i.e. calcified aorta, scarring from previous surgery). However, data from TAVI registries in many countries are very promising, and TAVI technique might represent one of the most important innovation in cardiac surgery of the last years [19–21].

With regards to mitral valve surgery, a recent systematic review about octogenarians reported that mortality risk is rather high (15%) [13]. However, the data clearly show that the operative risk has been markedly lower in the most recent series, as a consequence of the improvements in patient selection, surgical techniques, intraoperative management, and postoperative care. Indeed, we only observed a 6% mortality rate in mitral valve surgery. Furthermore, institutions with experience in valve repair, the mitral valve can be effectively reconstructed even in octogenarians, avoiding prosthesis-related problems. Using additional procedures only if strictly required may have positive influence on the patient’s outcome [22–24]. In our institution we proceed according to a strategy which aims at minimizing the surgery induced trauma (concomitant surgical procedures are possibly avoided and expeditious surgical technique of mitral valve repair is adopted) and at optimizing the treatment of postoperative hemodynamic instability and low output syndrome given the little tolerance to postoperative complications by the elderly. The impact of major complication (i.e. acute renal failure requiring renal replacement therapy, low output syndrome requiring IABP support and sepsis) on mortality in octogenarians is enormous: in this series, patients suffering from at least one of the major complications above had a mortality rate of 17%. In the elderly the potential benefits on organ function of new drugs (eg. levosimendan, fenoldopam) or mechanical circulatory support still has to be clarified. Moreover, many strategies have shown relevant benefits in cardiac surgery, but their role in this subset of patients has never been investigated: for example the use of postoperative non invasive ventilation or the use of volatile anesthetics for maintance of anesthesia were already proven to have positive effects on outcome and may be confirmed to have benefits also in octogenarians [25, 26].

In our center between 1998 and 2001 the octogenarians who underwent cardiac surgery were only 1.8% of the total, but we observed a very low mortality rate (1.8%).

In the light of these data, we expanded our experience in the following years with less strict contraindications to surgery, and people aged 80 or more represents nowadays 3.5% of the patients receiving cardiac surgery, with a reasonably low mortality (3.85%).

Despite these results, it is still an open issue whether the benefits, in terms of quality of life expectations after surgery, outbalance the risk in the elder. Gjeilo and colleagues observed an improvement in quality of life in patients aged 75 years or more from baseline to 6 months postoperatively, and remained relatively stable 5 years after cardiac surgery [27]. Khan and collaborators found that in most octogenarians hospital morbidity is increased, and hospital stay is longer [28]. On the contrary, Deschka et al. found that advanced age is correlated with a higher mortality, but not with prolonged ICU treatment or higher costs after cardiac surgery [29]. On the opposite, in a recent study by Meziere et al., age was found to be an independent risk factor of postoperative mortality and postoperative complications including cognitive dysfunction, with no impact of the choice of anesthetic technique on risk [30].

Notably, we were able to identify only 1 mortality risk predictor at baseline in this population: preoperative COPD, which is extremely common in the elderly. Therefore, the stratification of old patients candidate to undergo cardiac surgery is not trivial.

We acknowledge some limitations of our work. The study is retrospective and covers a long period of time during which the perioperative management of cardiac surgical patients may have been partially changed, both for young and old persons. We did not mention separately the postoperative complications due to infection because of lacking of complete data. We also could not provide data on diastolic dysfunction, which has a high prevalence in this population and could be extremely invalidating. Few definitions used in this paper (eg clinical condition at hospital discharge) are based on local protocols in the absence of universally accepted international criteria. We acknowledge also that the trial design did not include a follow-up. Furthermore, although we conclude that cardiac surgery may have benefits for the elderly, a score of the quality of life after surgery was not available for these patients.

## Conclusions

Clinicians should be strongly aware that cardiac surgery can be safely performed at all ages: meticulous preoperative evaluation is the key element which may help to stratify patients and discriminate those patients for whom surgery may be worthwhile (even if mortality predictors are few and aspecific). Hemodynamic treatment should be optimized in order to prevent major complications, as they may dramatically impair the outcome. In particular, delays and mistakes should be carefully avoided, as this population of patients is more fragile, and shows a limited physiologic reserve and resistance to any kind of stress. Discussion concerning all the therapeutic options with the patients and with the relatives is always mandatory.

## Electronic supplementary material

Additional file 1: **Comparison of all the variables between genders.** Preoperative evaluation. Comparison of all the variables presented in the manuscript between genders (male versus female). Preoperative checklist for evaluation of patients candidate to cardiac surgery at our institution. (DOC 145 KB)

## Abbreviations

AMI: Acute myocardial infarction
ACT: Activated clotting time
BMI: Body mass index
CABG: Coronary artery bypass grafting
COPD: Chronic obstructive pulmonary disease
CPB: Cardiopulmonary bypass
CVVH: Continuous venovenous hemofiltration
EF: Ejection fraction
FOP: Foramen ovale pervium
IABP: Intraaortic balloon pump
IAD: Interatrial defect
ICU: Intensive care unit
IVD: Interventricular defect
NYHA: New York Heart Association
TIA: Transient ischemic attack
TAVI: Transcatheter aortic valve implantation.
