# Supplementary material for: Cardiac surgery in 260 octogenarians: a case series
Source: BMC Anesthesiol. 2015 Jan 26;15(1):15. doi: 10.1186/1471-2253-15-15 (PMC4328195; doi:10.1186/1471-2253-15-15)
Supplement: Supplementary file 1 — Additional file 1: Comparison of all the variables between genders. Preoperative evaluation. Comparison of all the variables presented in the manuscript between genders (male versus female). Preoperative checklist for evaluation of patients candidate to cardiac surgery at our institution. (DOC 145 KB) [file 12871_2014_343_MOESM1_ESM.doc]

**Comparison of all the variables between genders.**

**Table S1.**

**Baseline characteristics.** Data are expressed as median (interquartile), mean ± standard deviation or number (percentage).

| **Variable** | **Female (N=130)** | **Male (N=130)** | **P-value** |
| --- | --- | --- | --- |
| Age, y | 82 ± 1.9 | 82 ± 1.7 | 0.9 |
| Height, cm | 160 ± 6.5 | 171 ± 6.1 | < 0.0001 |
| Weight, kg | 63 ± 11.3 | 74 ± 10.1 | < 0.0001 |
| BMI | 25.2 ± 3 | 24.5 ± 4 | 0.15 |
| **Comorbidity** |  |  |  |
| > COPD, n | 25 (19%) | 33 (25%) | 0.2 |
| > Preoperative EF, % | 60 (50 - 60) | 55 (45 - 60) | 0.2 |
| > Preoperative EF<=40%, n | 18 (15%) | 23 (20%) | 0.3 |
| > Preoperative EF<=30%, n | 15 (12%) | 21 (16%) | 0.3 |
| > Peripheral vasculopathy, n | 36 (28%) | 50 (38%) | 0.065 |
| > Hypertension, n | 74 (57%) | 72 (55%) | 0.8 |
| > Type II Diabetes, n | 15 (12%) | 17 (13%) | 0.7 |
| > Carotid stenosis, n | 17 (13%) | 26 (20%) | 0.13 |
| > Chronic pulmonary disease, n | 16 (12%) | 23 (18%) | 0.2 |
| > Angina, n | 9 (6.9%) | 22 (17%) | 0.013 |
| > Previous AMI, n | 12 (9.2%) | 26 (20%) | 0.014 |
| > Previous vascular surgery, n | 3 (2.3%) | 13 (10%) | 0.01 |
| > Euroscore standard | 9 (7 - 9) | 8 (7 - 9) | 0.059 |
| > Endocarditis, n | 1 (0.77%) | 4 (3.1%) | 0.4 |
| > Chronic renal failure, n | 10 (7.7%) | 22 (17%) | 0.024 |
| > Dialysis, n | - | - | - |
| Charlson Comorbidity Index | 5.8 ± 1.12 | 6.2 ± 1.39 | 0.008 |
| **NYHA class** |  |  | 0.03 |
| > I, n | 7 (7.5%) | 8 (9.4%) |  |
| > II, n | 34 (36.6%) | 48 (56.5%) |  |
| > III, n | 48 (51.6%) | 26 (30.6%) |  |
| > IV, n | 4 (4.3%) | 3 (3.5%) |  |
| **Timing of surgery** |  |  | 0.2 |
| > Emergency, n | 2 (1.6%) | 3 (2.3%) |  |
| > Urgency, n | 12 (9.2%) | 21 (16.2%) |  |
| > Planned, n | 116 (89.2%) | 106 (81.5%) |  |
| Redo, n | 4 (3.1%) | 7 (5.4%) | 0.4 |
| Previous cardiac surgery with CPB, n | 2 (1.5%) | 3 (2.3%) | 0.9 |
| Previous cardiac surgery without CPB, n | 1 (0.77%) | 1 (0.77%) | 0.9 |
| Preoperative IABP, n | 4 (3.1%) | 1 (0.77%) | 0.4 |
| Chronic Therapy |  |  |  |
| > Antiplatelets, n | 46 (35%) | 51 (39%) | 0.5 |
| > Diuretics, n | 80 (62%) | 61 (47%) | 0.018 |
| > Betablockers, n | 45 (35%) | 50 (38%) | 0.5 |
| > Antibiotics, n | 1 (0.77%) | 4 (3.1%) | 0.4 |
| > Ca-channels antagonists, n | 26 (20%) | 29 (22%) | 0.6 |
| > Nitrates, n | 23 (18%) | 43 (33%) | 0.004 |
| > ACE- inhibitors, n | 80 (62%) | 71 (55%) | 0.3 |
| > Oral anticoagulants, n | 21 (16%) | 17 (13%) | 0.5 |
| > Heparin, n | 4 (3.1%) | 14 (11%) | 0.015 |
| Bilirubine, mg/dl | 0.83 ± 0.43 | 0.84 ± 0.38 | 0.6 |
| Creatinine, mg/dl | 0.94 ± 0.27 | 1.19 ± 0.47 | < 0.0001 |

BMI – body mass index; COPD – chronic obstructive pulmonary disease; EF – ejection fraction; AMI – acute myocardial infarction; TIA – transient ischemic attack; NYHA – New York Heart Association; CPB – cardiopulmonary bypass; IABP – intraaortic balloon pump.

**Table 2.**

**Operation type.** Data are expressed as number (percentage).

| **Variable** | **Female (N=130)** | **Male (N=130)** | **P-value** |
| --- | --- | --- | --- |
| CABG, n of patients | 49 (38%) | 70 (54%) | 0.009 |
| > Isolated CABG, n of patients | 16 (12%) | 47 (36%) | < 0.0001 |
| Mitral valve surgery, n | 40 (31%) | 27 (21%) | 0.065 |
| > Isolated mitral valve surgery, n | 10 (7.7%) | 9 (6.9%) | 0.8 |
| > Mitral valve replacement, n | 21 (16%) | 8 (6.2%) | 0.01 |
| > Mitral valve repair, n | 19 (15%) | 19 (15%) | 0.9 |
| Aortic valve surgery, n | 80 (62%) | 52 (40%) | 0.0005 |
| > Isolated aortic valve surgery, n | 43 (33%) | 28 (22%) | 0.037 |
| > Aortic valve repair, n | 1 (0.77%) | 0 (0%) | 0.9 |
| > Aortic valve replacement, n | 79 (61%) | 52 (40%) | 0.0008 |
| Tricuspid valve surgery, n | 19 (15%) | 15 (12%) | 0.5 |
| > Isolated tricuspid valve surgery, n | 0 (0%) | 1 (0.77%) | 0.9 |
| > Tricuspid valve replacement, n | 1 (0.77%) | 0 (0%) | 0.9 |
| > Tricuspid valve repair, n | 18 (14%) | 15 (12%) | 0.6 |
| Ascending aorta surgery, n | 14 (11%) | 11 (8.5%) | 0.5 |
| > Isolated ascending aorta surgery, n | 1 (0.77%) | 1 (0.77%) | 0.9 |

CABG – coronary artery bypass graft; IVD – interventricular defect; IAD – interatrial defect; FOP – foramen ovale pervium.

**Table 3.**

**Intraoperative data. Data are expressed as median (interquartile), mean ± standard deviation or number (percentage).**

| **Variable** | **Female (N=355)** | **Male (N=436)** | **P-value** |
| --- | --- | --- | --- |
| CPB | 122 (94%) | 114 (88%) | 0.087 |
| Duration of aortic clamping, min | 62 ± 22.1 | 61 ± 22.4 | 0.7 |
| Duration of CPB, min | 79 ± 26.5 | 81 ± 25.1 | 0.6 |
| Bleeding in the first 12 postoperative hours, ml | 200 (150 - 330) | 250 (190 - 380) | 0.007 |
| Total postoperative bleeding, ml | 325 (200 - 465) | 390 (250 - 600) | 0.08 |
| Creatinine peak, mg/dl | 1.06 ± 0.43 | 1.4 ± 0.69 | < 0.0001 |
| Acute kidney injury, n | 20 (15%) | 27 (21%) | 0.3 |
| CVVH, n | 5 (3.9%) | 3 (2.3%) | 0.7 |
| Patients receiving hemoderivates, n | 69 (53%) | 41 (32%) | 0.0004 |
| RBC transfusions, n of units per patient | 1 (0 - 2) | 0 (0 - 1) | <0.0001 |
| FFP transfusions, n of units per patient | 0 (0 - 0) | 0 (0 - 0) | 0.6 |
| PLT transfusions, n of units per patient | 0 (0 - 0) | 0 (0 - 0) | 0.094 |
| Neurologic damage type 1, n | 2 (1.5%) | 0 (0%) | 0.5 |
| Neurologic damage type 2, n | 3 (2.3%) | 4 (3.1%) | 0.9 |
| Postoperative AMI, n | 1 (0.77%) | 3 (2.3%) | 0.6 |
| Troponine peak, ng/ml | 6.9 (4.6 - 11.3) | 7.8 (4 - 13.3) | 0.6 |
| Atrial fibrillation, n | 38 (29%) | 32 (25%) | 0.4 |
| Duration of mechanical ventilation, hours | 15 (12 - 20) | 15 (11 - 21) | 0.6 |
| Severe pulmonary dysfunction, n | 2 (1.5%) | 3 (2.3%) | 0.9 |
| Mild pulmonary dysfunction, n | 10 (7.7%) | 6 (4.6%) | 0.3 |
| Tracheostomy, n | 5 (3.9%) | 2 (1.5%) | 0.4 |
| Need for re-intubation, n | 3 (2.3%) | 3 (2.3%) | 0.9 |
| Low cardiac output syndrome, n | 21 (16%) | 23 (18%) | 0.9 |
| Cardiogenic shock, n | 0/260 | 6 (4.6%) | 3 (2.3%) |
| Inotropes for more than 48 hours, n | 17 (13%) | 19 (15%) | 0.7 |
| Sepsis, n | 2 (1.5%) | 2 (1.5%) | 0.9 |
| Mediastinitis, n | - | - | - |

CPB – cardiopulmonary bypass; CVVH – continous venovenous hemofiltration; IABP – intraaortic balloon pump; AMI – acute myocardial infarction; ICU – intensive care unit.

**Table 4.**

**Outcomes. Data are expressed as median (interquartile), mean ± standard deviation or number (percentage).**

| **Variable** | **Female (N=130)** | **Male (N=130)** | **P-value** |
| --- | --- | --- | --- |
| Hospital mortality, n | 7 (5.4%) | 3 (2.3%) | 0.2 |
| ICU stay, days | 2 (1 - 4) | 2 (1 - 3) | 0.14 |
| Hospital stay, days | 7 (5 - 9) | 6 (5 - 8) | 0.2 |
| Clinical condition at hospital discharge |  |  | 0.6 |
| > Dead, n | 7 (5.4%) | 3 (2.3%) |  |
| > Extremely poor, n | 1 (0.8%) | 1 (0.8%) |  |
| > Poor, n | 4 (3.1%) | 7 (5.4%) |  |
| > Good, n | 118 (90.7%) | 119 (91.5%) |  |

ICU – intensive care unit.

**Preoperative evaluation.**

**Table 5.** A senior cardiac surgeon evaluates the patients on an ambulatory basis approximately 1 month before surgery. A junior cardiac surgeon re-evaluate the patient on the day of hospital admission. A senior anesthesiologist evaluate the patient on the day before surgery (usually immediately after the junior surgeon evaluation). The data in this table are available for all patients before surgery. Additional information or examinations are performed whenever deemed appropriate in selected patients and might slightly delay the day of surgery. The final decision to operate the patient is taken in the afternoon of the day before surgery. A final morning surgical meeting on the day of surgery permits to summarize all the findings and decide and coordinate intraoperative strategies.

| Demographic information | - |
| --- | --- |
| Baseline parameters | Height  Weight  Allergies  Smoke (past or active) |
| Comorbidities and illness | Past and/or present |
| Previous surgery | Including type of anesthesia (and eventual problems) |
| Airway evaluation | - |
| Functional status | NYHA class |
| Electrocardiagram | - |
| TE echocardiography | - |
| Coronary angiography | . |
| Chest Xray | - |
| Routine blood tests | Hemocrome, Electrolytes, Prothrombin time, Activated partial thromboplastin time, Serum glucose, Serum creatinine, Urea, Aspartate aminotransferase, Alanin aminotransferase, Gamma-glutamyl transferase, Bilirubin, Creatine phosphokinase, Troponine, Lactate dehydrogenase, Alkaline phosphatase, Plasma proteins, C reactive protein, Type and screen, blood group, Pregnancy test (for women under 50 years) |
| ASA physical status classification |  |
| Therapy | Chronic and present.  Long half life ACEI are suspended or switched to captopril on hospital admission. |

TE – transesophageal, ASA – American Society of Anesthesiologists, ACEI – Angiotensin converting enzyme inhibitors.
